# Exploring the effect of alcohol on disease activity and outcomes in rheumatoid arthritis through systematic review and meta-analysis

**DOI:** 10.1038/s41598-021-89618-1

**Published:** 2021-05-18

**Authors:** Jaime N. Turk, Erin R. Zahavi, Aine E. Gorman, Kieran Murray, Matthew A. Turk, Douglas J. Veale

**Affiliations:** 1 Queen’s University, Kingston, ON Canada; 2 Department of Rheumatology, Saint Vincent’s University Hospital, Dublin 4, Ireland; 3 EULAR Centre for Arthritis and Rheumatic Diseases, Dublin Academic Medical Centre, Dublin, Ireland

**Keywords:** Rheumatic diseases, Risk factors

## Abstract

To evaluate the effects of alcohol consumption on disease activity in rheumatoid arthritis. EMBASE, Pubmed, the Cochrane Library, and Web of Science were searched until July 29, 2020. English language studies that reported disease activity outcomes in rheumatoid arthritis were included. Studies were excluded if they were reviews, case reports, had fewer than 20 patients, or reported on prevalence but not disease activity in RA. Forest plots were used to determine pooled mean difference and were generated on RevMan5.3. Linear regression was used to determine correlations between alcohol and antibody status, gender, and smoking status. The search identified 4126 citations of which 14 were included. The pooled mean difference in DAS28 (95% CI) was 0.34 (0.24, 0.44) (*p* < 10^−5^) between drinkers and non-drinkers with lower DAS28 in non-drinkers, 0.33 (0.05, 0.62) (*p* = 0.02) between heavy drinkers and non-drinkers with lower DAS28 in heavy drinkers, and 0.00 (− 0.30, 0.30) (*p* = 0.98) between low- and high-risk drinkers. The mean difference of HAQ assessments was significantly different between those who drink alcohol compared to those who do not, with drinkers reporting lower HAQ scores (0.3 (0.18, 0.41), *p* < 10^−5^). There was no significant correlation between drinking and gender, smoking status, or antibody positivity. Alcohol consumption is associated with lower disease activity and self-reported health assessment in rheumatoid arthritis. However, drinking has no correlation with smoking, gender, or antibody status.

## Introduction

Rheumatoid Arthritis (RA) is a chronic inflammatory condition which if left untreated can lead to joint inflammation, damage and a reduction in life expectancy^1,2^. Both genetic and environmental risk factors contribute to the aetiology of RA with their impact varying depending on a patient’s rheumatoid factor (RF) and whether they have antibodies to citrullinated protein antigen (ACPA)^3,4^. The most recognised environmental risk factor associated with RA is smoking^5^. Studies have shown that smoking increases the risk of developing ACPA positivity as well as decreases response to treatment^6–8^. Other environmental risk factors which have been studied in relation to the development of RA include alcohol intake, diet, vitamin D and education levels^9–12^. However, the impact of these risk factors on both the development and severity of RA remains unclear.

Several studies have assessed the role of alcohol consumption and its association with the risk of developing RA, with many suggesting that alcohol is associated with the incidence of RA^13–17^. A previous meta-analysis investigating the protective effect of alcohol on developing RA showed alcohol intake was inversely associated with ACPA-positive RA, proposing a protective effect^18^.

The influence of environmental factors on disease activity is less understood. Alcohol has been shown to downregulate the synthesis of pro-inflammatory cytokines and in mouse models it has been associated with a reduction in joint destruction and disease activity^14,19,20^. In a Swedish study, alcohol consumption is shown to be associated with a reduction in disease activity in women but not men^15^. Another study suggests that frequency of alcohol consumption is inversely associated with radiological damage and disease activity^13^. However, other studies suggest that alcohol has no association with disease activity^21^. This study presents a systematic review and a meta-analysis to evaluate the relationship between alcohol intake and disease activity in RA.

## Methods

### Study selection

The PICO (population, intervention, control, and outcomes) methodology was used to generate the research question comparing the mean difference in disease activity in RA patients with different levels of alcohol consumption. Search terms were generated using exploded search terms on EMBASE for topics pertaining to alcohol consumption in RA and are listed in “Appendix 1”. EMBASE, Pubmed, the Cochrane Library, and Web of Science were searched from their inceptions until July 29, 2020. Studies reporting on alcohol consumption and disease activity in a cohort of RA patients were included for further investigation.

### Inclusion criteria and data extraction

English language studies were included if they presented disease activity outcome measures in patients with RA including: the disease activity score based on a 28 joint count (DAS28), C-reactive protein (CRP), erythrocyte sedimentation rate (ESR), tender joint count (TJC), swollen joint count (SJC), rheumatoid factor (RF) positivity, Anti-citrullinated protein antibody (ACPA) positivity, and/or health assessment questionnaire (HAQ). Observational studies that were either longitudinal or cross sectional were included. Studies were excluded if they were case reports or review articles, if they had fewer than 20 patients or if they reported on the prevalence of RA, not its disease activity. The strengthening the reporting of observational studies in epidemiology (STROBE) checklist was used to evaluate individual study bias^22^. Data were extracted from baseline assessment of alcohol consumption.

### Statistical analysis

RevMan5.3 was used to generate forest plots from 95% confidence intervals of extracted data using mean differences. I-squared and tau-squared statistics were used to evaluate variance and heterogeneity across included studies. Random effects models were used to generate forest plots. Linear regression was used on SPSS26 to evaluate correlations between alcohol consumption and other factors including proportions of smoking, serology, and gender within each cohort.

### Ethics approval and consent to participate

None was needed as it is a systematic review.

## Results

**Figure 1:**
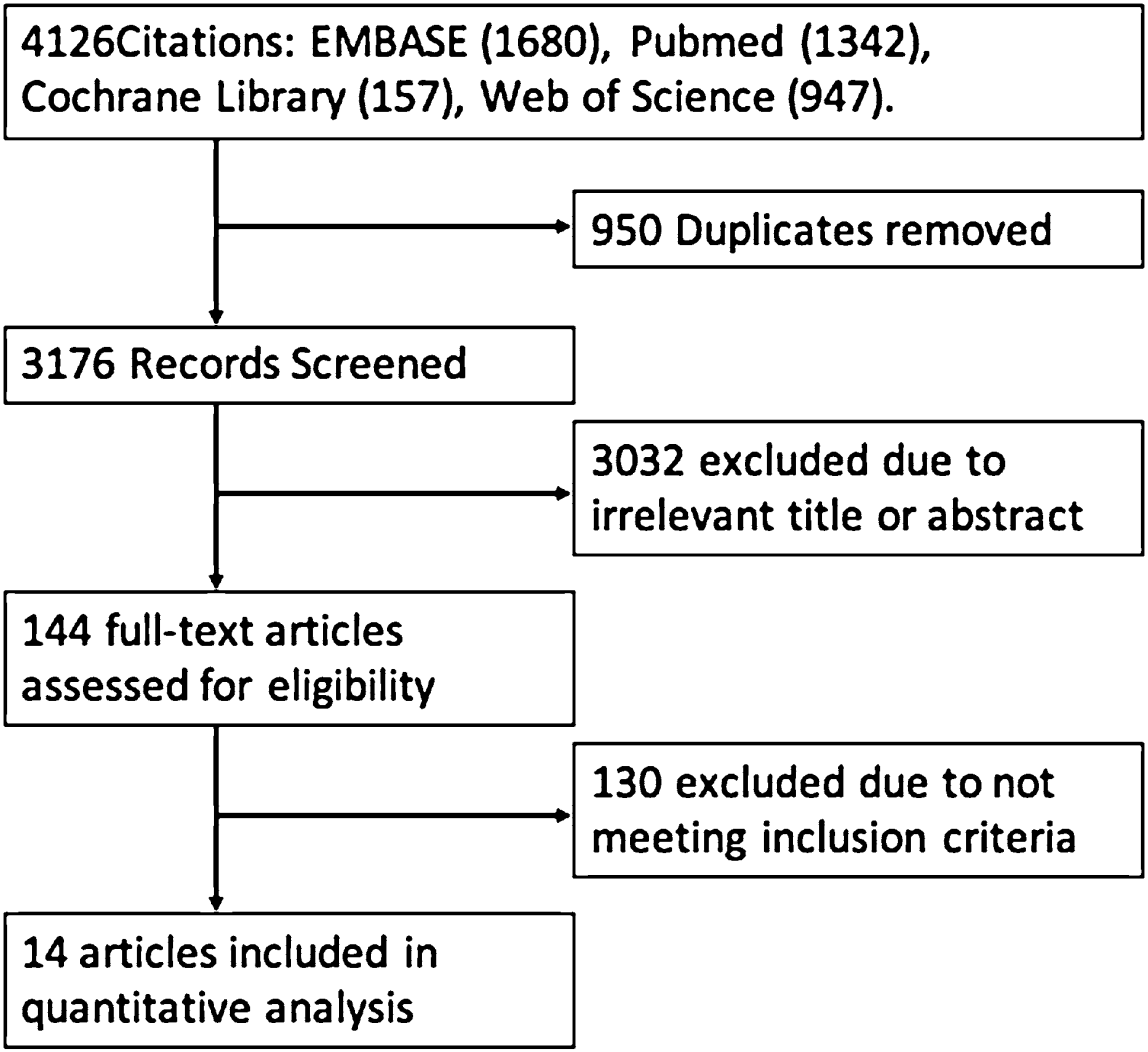
PRISMA flow of data collection.

The literature search identified 4126 papers of which 14 were included for analysis (Fig. 1, Table 1)^23^. These papers represented data from 16,347 patients. Heterogeneity, according to the I^2^ statistic, was between 97 and 99% and thus a random effects model was used to calculate pooled mean differences and prevalence.

**Table 1:** Studies analysed.

First author (citation)	Year	Country	n	Proportion of patients who drink	Proportion of patients who are female	Proportion of patients who smoke	Strobe Score
Bergman^15^	2013	Sweden	1238	0.887	0.7	0.17	18
Bergstrom^9^	2013	Sweden	172	0.894	0.791	0.385	18
Bing^16^	2013	US	615	0.666	0.829	0.0846	18
Gunter^24^	2017	S. Africa	177	0.316	0.825	0.102	16
Larsson^25^	2018	Sweden	1509	0.904	0.71	Not reported	19
Mangnus^26^	2018	Netherlands	380	0.644	0.644	0.284	19
Marshall^27^	2013	US	166	0.608	0.855	0.259	20
Maxwell^13^	2010	UK	873	0.708	0.722	0.594	19
Nissen^28^	2010	Switzerland	2908	0.627	0.7542	0.2666	18
Pala^29^	2013	US	2764	0.403	0.816	Not reported	N/A
Sageloli^30^	2018	France	596	0.173	0.775	0.209	15
Shimizu^31^	2017	Japan	4695	0.506	0.866	Not reported	N/A
Taşpınar^32^	2018	Turkey	56	0.089	0.84	0.375	15
Yoshimura^33^	2019	Japan	198	0.364	0.843	0.298	N/A

**Figure 2:**
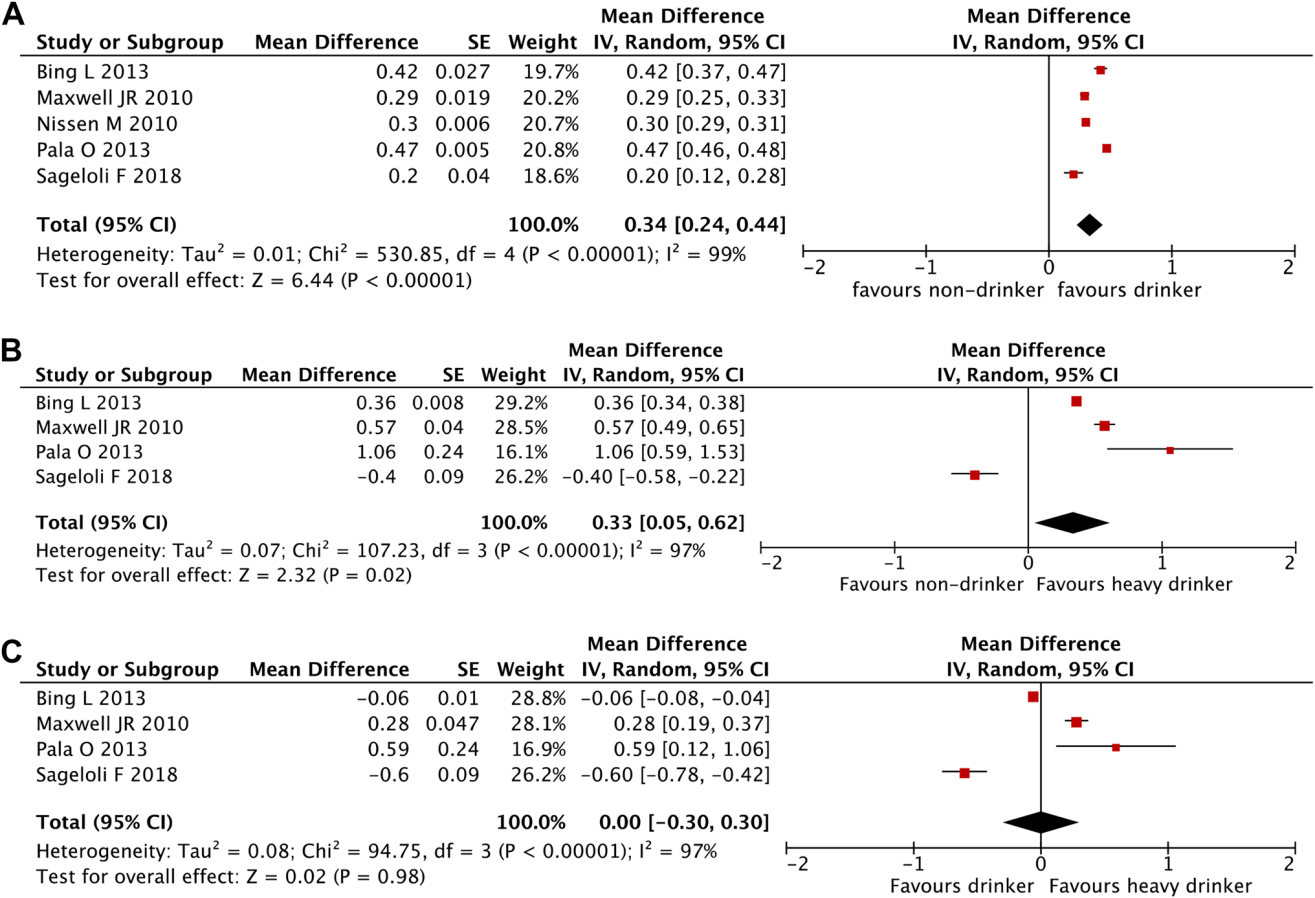
Mean differences in DAS28 between drinking groups. (**A**) Between non-drinkers and drinkers. (**B**) Between non-drinkers and high-risk drinkers. (**C**) Between low-risk and high-risk drinkers.

There was significantly lower disease activity in those who drink alcohol compared to those who do not (Fig. 2). The pooled mean difference in disease activity (95% CI) according to the DAS28 was 0.34 (0.24, 0.44) between non-drinkers and drinkers (*p* < 10^−5^) with non-drinkers having a higher mean DAS28. Between non-drinkers and heavy drinkers, the pooled mean difference in DAS28 was 0.33 (0.05, 0.62) (*p* = 0.02) with non-drinkers having a higher mean DAS28. Among those who drink, the mean difference between low-risk drinkers and high-risk drinkers was 0 (− 0.3, 0.3) (*p* = 0.98).

**Figure 3:**
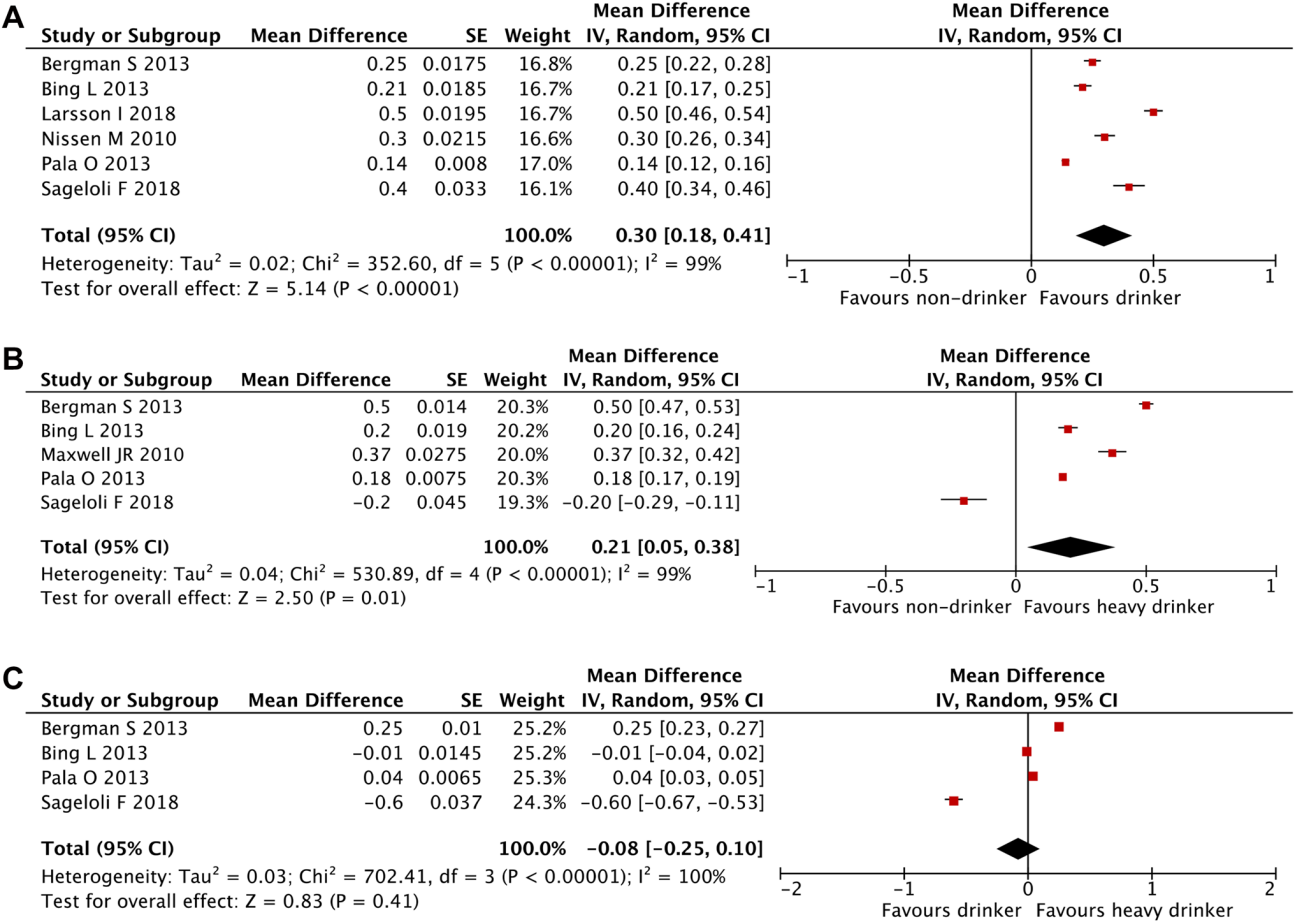
Mean differences in HAQ between drinking groups. (**A**) Between non-drinkers and drinkers. (**B**) Between non-drinkers and high-risk drinkers. (**C**) Between low-risk and high-risk drinkers.

There was also a significant difference in HAQ between non-drinkers and drinkers with those who drink having lower HAQ scores (Fig. 3). The pooled mean difference in HAQ between non-drinkers and drinkers was 0.3 (0.18, 0.41) (*p* =  < 10^−5^) whereby non-drinkers had higher HAQ scores. Between non-drinkers and heavy drinkers, the pooled mean difference in HAQ was 0.21 (0.05, 0.38) (*p* = 0.01) with non-drinkers having higher HAQ scores. Among those who drink, the mean difference between low-risk drinkers and high-risk drinkers was − 0.08 (− 0.25, 0.1) (*p* = 0.41).

When analyzing the consumption habits of the cohorts, the proportion of those who drank was 0.55 (0.44, 0.67). The total pooled proportion of the cohorts considered high risk drinkers was 0.25 (0.14, 0.35).

Across cohorts, gender (*p* = 0.057) and smoking status (*p* = 0.734) were not correlated to patient drinking status. High risk drinking was weakly correlated with male gender (R^2^ = 0.0027, *p* = 0.022) but was not correlated with smoking status (*p* = 0.59). RF positivity (*p* = 0.424, R^2^ = 0.22) and ACPA positivity (*p* = 0.230, R^2^ = 0.59) were not significantly associated with increases in disease activity.

STROBE scores for studies ranged between 15 and 20. CRP, ESR, and joint counts were not correlated with alcohol consumption.

## Discussion

Our systematic analysis supported a relationship between alcohol consumption and disease activity in RA, where disease activity and HAQ scores are lower in those who consume alcohol compared to those who do not. Despite no significant difference seen between levels of alcohol consumption, both HAQ and DAS28 CRP were significantly lower in non-drinkers compared to those who consume alcohol.

Previous studies in Sweden and the UK have shown that moderate alcohol intake was associated with lower disease activity and improved quality of life measures^15^. A Swedish study showed that there was an association between alcohol consumption and better health related quality of life in women but not in men^15^. Measures of disease activity including the modified HAQ were found to be inversely proportional to alcohol consumption in both men and women in the 873 patients with RA in the United Kingdom^15^. This association was also present in our systematic review, with lower HAQ scores in patients who drink compared to non-drinkers.

Some studies have suggested an inverse relationship between alcohol consumption and RA development, which is predominately related to ACPA positive patients^13,18^. However, when looking at the ACPA status, alcohol consumption and disease activity of patients, there was no correlation between APCA positive patients’ alcohol consumption and disease activity. ACPA status was inconsistently reported in a portion of evaluated studies, therefore caution when interpreting results is required. A previous meta-analysis suggests an inverse relationship between alcohol consumption and the development of RA in ACPA positive individuals^18^. ACPA positive RA has been associated with environmental risk factors including smoking status and alcohol intake^4^. Smoking has been associated with more aggressive disease and poorer outcomes in patients with RA^34,35^. In this study, there was no relationship between alcohol intake and smoking amongst RA patients.

Strengths of this work include its robust search strategy. A broad search was conducted across four databases to identify all possible citations and included both abstracts and conference presentations. The double extraction protocol used for article selection minimizes selection bias and allows for a thorough evaluation. While the heterogeneity of studies was high, a random effects model to calculate pooled statistics was used. This model relaxes the assumption that the true relationship between alcohol consumption and disease activity does not vary between studies, however it does not alone control for confounding^36^. Therefore, regression was performed to assess the relationship between alcohol consumption and other demographic information such as gender, smoking status, and RF/ACPA positivity. Since these studies were mostly cross sectional, age could not be meaningfully assessed in regression models.

Our study is not without limitations. Individual studies categorize drinking habits differently and different regions have varying standards for what they consider to be high-risk drinking. In the UK, men who consume more than 14 standard drinks and women who consume more than 10 per week are considered to be at risk^37^. We did not differentiate between the different international standards for drinking risk within our analysis as the breadth of literature to do so was unavailable. The mean age also varied between cohorts, which could have an influence on alcohol consumption. However, in linear regression models, gender, smoking status, and antibody status had no relationship with alcohol consumption in RA. The study could also be susceptible to the ecological fallacy, each study may have its own unique relationship that may be diluted when making pooled comparisons. There could be confounding variables which potentially mask further differences seen in the individual studies^38^. In addition, studies that met the inclusion criteria were all conducted after 2010. This is coincidental as older studies did not consistently compare groups for analysis.

Overall, there is an association with alcohol consumption and better disease activity scores in patients with RA. The pooled proportion of those with RA who drink alcohol in these cohorts is 56%. When comparing demographic data to alcohol consumption there is no significant correlation between alcohol use and smoking nor gender. In addition, no correlation between alcohol consumption and antibody status was observed.

## Supplementary information

Supplementary Information.

## Data Availability

The datasets used and analysed during the current study are available from the corresponding author on reasonable request.
