# Supplementary material for: Exploring the effect of alcohol on disease activity and outcomes in rheumatoid arthritis through systematic review and meta-analysis
Source: Sci Rep. 2021 May 18;11:10474. doi: 10.1038/s41598-021-89618-1 (PMC8131728; doi:10.1038/s41598-021-89618-1)
Supplement: Supplementary file 1 — Supplementary Information. [file 41598_2021_89618_MOESM1_ESM.docx]

**Exploring the effect of alcohol on disease activity and outcomes in rheumatoid arthritis through systematic review and meta-analysis**

**Appendix 1**

Jaime N Turk^1^, Erin R Zahavi^1^, Aine E Gorman^2,3^, Kieran Murray^2,3^, Matthew A Turk^2,3^, and Douglas J Veale^2,3^

1 Queens university, Kingston Ontario Canada

2 Department of Rheumatology, Saint Vincent’s University Hospital, Dublin 4, Ireland

3 EULAR Centre For Arthritis and Rheumatic Diseases, Dublin Academic Medical Centre, Dublin, Ireland

**Correspondence:** 17jnt@queensu.ca, 17erz@queensu.ca, ainegorman1@gmail.com, kemurray@hotmail.com, mturk5@uwo.ca, douglas.veale@ucd.ie

**Ethics approval and consent to participate:** none was needed as it is a systematic review

**Consent for publication:** Not applicable

**Availability of data and materials:** The datasets used and analysed during the current study are available from the corresponding author on reasonable request.

**Competing interests:** The authors report no conflicts of interests

**Funding:** The centre for arthritis and rheumatic disease

**Authors' contributions:** JNT and ERZ performed the literature search, data extraction, and were involved with manuscript writing. AEG, KM, and MAT were involved with statistical analysis, study design, and manuscript writing. DJV was involved with study design and manuscript writing.

**Acknowledgements:** We’d like to thank the professional and research staff at the bone and joint unit and centre for rheumatic disease at St. Vincent’s University hospital, Dublin 4, Ireland.

**Appendix 1. search strategy**

| Rheumatoid arthritis  (OR) | (AND) | Alcohol  (OR) |
| --- | --- | --- |
| Inflammatory Arthritis |  | alcohol |
| arthritis deformans |  | drinking alcohol |
| arthritis, rheumatoid |  | excessive drinking |
| arthrosis deformans |  | hazardous drinking |
| beauvais disease |  | problem drinking |
| chronic polyarthritis |  | problematic drinking |
| chronic progressive poly arthritis |  | alcohol consumption |
| chronic progressive polyarthritis |  | alcohol intake |
| chronic rheumatoid arthritis |  | heavy drinking |
| disease, beauvais |  | alcohol abuse |
| infantile rheumatoid arthritis |  | abuse, alcohol |
| inflammatory arthritis |  |  |
| polyarthritis, primary chronic |  |  |
| primary chronic polyarthritis |  |  |
| progressive polyarthritis, chronic |  |  |
| rheumarthritis |  |  |
| rheumatic arthritis |  |  |
| rheumatic polyarthritis |  |  |
| rheumatism, chronic articular |  |  |

**Figure 1 supplementary. Funnel plot analysis of publication bias of papers reported**
